# Emotional discrimination during viewing unpleasant pictures: timing in human anterior ventrolateral prefrontal cortex and amygdala

**DOI:** 10.3389/fnhum.2015.00051

**Published:** 2015-02-10

**Authors:** Satoru Kohno, Madoka Noriuchi, Yoshinobu Iguchi, Yoshiaki Kikuchi, Yoko Hoshi

**Affiliations:** ^1^Tokyo Metropolitan Institute of Medical ScienceTokyo, Japan; ^2^Graduate School of Tokyo Metropolitan UniversityTokyo, Japan

**Keywords:** functional magnetic resonance imaging (fMRI), IAPS pictures, amygdala, emotion, emotional discrimination, ventrolateral prefrontal cortex, timing estimation

## Abstract

The ventrolateral prefrontal cortex (VLPFC) and amygdala have critical roles in the generation and regulation of unpleasant emotions, and in this study the dynamic neural basis of unpleasant emotion processing was elucidated by using paired-samples permutation *t*-tests to identify the timing of emotional discrimination in various brain regions. We recorded the temporal dynamics of blood-oxygen-level-dependent (BOLD) signals in those brain regions during the viewing of unpleasant pictures by using functional magnetic resonance imaging (fMRI) with high temporal resolution, and we compared the time course of the signal within the volume of interest (VOI) across emotional conditions. Results show that emotional discrimination in the right amygdala precedes that in the left amygdala and that emotional discrimination in both those regions precedes that in the right anterior VLPFC. They support the hypotheses that the right amygdala is part of a rapid emotional stimulus detection system and the left amygdala is specialized for sustained stimulus evaluation and that the right anterior VLPFC is implicated in the integration of viscerosensory information with affective signals between the bilateral anterior VLPFCs and the bilateral amygdalae.

## Introduction

The human brain contains neural circuits responsible for the emotional responses elicited by stimuli, and the amygdala and prefrontal cortex (PFC) are key components of these circuits (Davidson and Irwin, 1999). The bilateral ventrolateral prefrontal cortices (VLPFCs) are involved, through the amygdalae, in both the generation and regulation of unpleasant emotions (Wager et al., 2008; Hoshi et al., 2011). Furthermore, recent functional magnetic resonance imaging (fMRI) studies have shown functional connectivity between the VLPFC and the amygdala (Banks et al., 2007; Guyer et al., 2008; Tang et al., 2013; Townsend et al., 2013). For instance, when subjects reappraise unpleasant scenes in order to decrease the strength of the unpleasant feelings elicited, VLPFC activity is inversely correlated with amygdala activity (Ochsner et al., 2002; Wager et al., 2008). In contrast, attenuated VLPFC function and/or heightened amygdala activation has been found in manic bipolar patients and high-anxiety adolescents when they were compared with healthy subjects (Foland et al., 2008; Guyer et al., 2008). Although fMRI research on functional connectivity has clarified temporal correlations between the activity an unpleasant picture stimulus elicits in the VLPFC and the activity it elicits in the amygdala, the dynamic neural basis of unpleasant emotion processing in these regions remains unclear.

Recently, our parametric mediation analysis of the activity affective pictures elicit in the VLPFCs, amygdalae, visual cortex, premotor cortex, supplementary motor area, fusiform gyrus and dorsolateral PFC found the activities in these regions to be negatively related with the valence ratings of affective pictures and found that the right anterior VLPFC (BA47) was only a mediator of the valence ratings, while the other brain regions were both sources and mediators (Kida et al., 2012). It has been widely accepted that the anterior VLPFC receives several kinds of sensory input (olfactory, gustatory, visceral, somatic, and visual) as well as limbic inputs from the amygdala, entorhinal and perirhinal cortex, and subiculum (Price, 1999). The right anterior VLPFC has therefore been hypothesized to be implicated in the integration of viscerosensory information with affective signals (Lévesque et al., 2004). It has also been hypothesized that the right amygdala is part of a rapid or dynamic system for detecting emotional stimuli and the left amygdala is specialized for sustained stimulus evaluation (Wright et al., 2001). If the right VLPFC plays a role in integrating emotional information, emotional discrimination in the right anterior VLPFC would occur after that in other brain regions. If the right amygdala were part of a rapid emotional stimulus detection system, the emotional discrimination there would precede that in the left amygdala. Here, emotional discrimination means regional brain reacts to unpleasant stimuli differently from neutral stimuli according to the study by Sabatinelli et al. (2009).

In the present study we examined the timing of emotional discrimination in the bilateral VLPFCs and the bilateral amygdalae in order to test the above-mentioned hypotheses about the roles of these brain regions in unpleasant emotion processing. We did this by focusing on three key brain regions—the visual cortex, amygdala and VLPFC—and using fMRI with high temporal resolution: 300 ms. Though the blood-oxygen-level-dependent (BOLD) signal in fMRI is delayed relative to the neural response, the timing of the signal within active regions is highly reliable (Kim et al., 1997; Menon and Kim, 1999; Miezin et al., 2000). Comparing the time course of the signal within the volume of interest (VOI) across emotional conditions eliminated potential confounding factors regarding the individual differences in BOLD signal timing (Aguirre et al., 1998; Buxton et al., 1998) and variations in vascular anatomy (Sabatinelli et al., 2009). Furthermore, the time course in the primary visual cortex was also investigated in order to verify the validity of our results.

## Materials and methods

### Subjects

Nineteen right-handed healthy volunteers (13 male, 6 female, mean age 20.4 ± 1.8 years) participated in the present study. None had a history of neurological or psychiatric disorders, and written informed consent was obtained from all of them. The ethics committee of the Tokyo Metropolitan Institute of Medical Science approved the study.

### Stimuli and procedure

To select appropriate stimuli, we had the emotional valence of 137 pictures from the International Affective Picture System (IAPS; Lang et al., 2008) and 100 pictures from other photograph collections (Fuji Film Co., Tokyo, Japan) rated by 33 healthy volunteers (12 male, 21 female, 20–28 years old), none of which was a subject in the present study (Hoshi et al., 2011). The emotional valence was rated on a digital scale with nine grades ranging from 1 (very unpleasant) to 9 (very pleasant).

**Figure 1:**
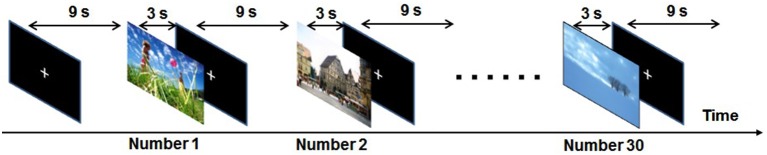
**Schematic diagram of the experimental paradigm in one run**. During each run 30 picture stimuli (10 unpleasant, 10 neutral and 10 pleasant) were randomly presented for 3.0 s and each was followed by a 9.0-s white cross-hair in the middle of the screen.

Ninety pictures for which the subjects’ ratings coincided were selected as stimuli: 30 unpleasant pictures (IAPS image numbers 3170, 1275, 9561, 7380, 9400, 3500, 1111, 1300, 9313, 2800, 8230, 9570, 9008, 9410, 7360, 3301, 3350, 9300, 3400, 9405, 1280, 2095, 9290, 1090, 3160, 6313, 9440, 3180, 6560 and 9007), 30 neutral pictures (IAPS image numbers 7025, 7041, 7090, 7100, 7140, 7115, 7211, 7009, 7705, 7004, 7150, 7235, 6150, 7006, 7503, 7224, 7233, 7950, 7010, 2221, 7034, 2520, 7000 and 5500) and 30 pleasant pictures (IAPS image numbers 1710, 1027, 1040, 2091, 2023, 1440, 7325, 2025 and 8005) (Hoshi et al., 2011). The “rating coincided” means that 33 volunteers rated a picture at the same valence range (1–3, 4–6, 7–9). The selected pictures included content such as mutilation, threatening animals, angry people, pollution, accidents, grief, nature, pets, children, neutral objects and neutral people. Erotic pictures were excluded because sex differences in the emotions they evoke have been seen (Sabatinelli et al., 2004). Experimental runs consisted of 30 picture presentations, and bias was avoided by randomly presenting 10 unpleasant, 10 neutral and 10 pleasant during each run. Each picture was presented for 3.0 s and was followed by a 9.0-s presentation of a white crosshair in the middle of the screen (Figure 1). As the purpose of our study was to investigate emotional discrimination during viewing unpleasant pictures, only unpleasant and neutral pictures (controls) might have been sufficient. However, to reduce psychological and neural effects of anticipation, we adopted a multiple randomization method, in which pleasant pictures were also presented in addition to unpleasant and neutral pictures. Under the experimental conditions, it is difficult for subjects to predict which kind of stimuli will be presented. Furthermore, in the present study, differences in BOLD signals between unpleasant and neutral stimuli were examined, which is expected to effectively reduce anticipation-related brain activations. Our fMRI experiment consisted of three runs, each using a different set of pictures. After the runs the subjects rated the emotional valence of each of the 90 pictures, as experienced at the time of the initial presentation, on a scale ranging from 1 (very unpleasant) to 9 (very pleasant) using Self-Assessment Manikin (SAM) rating methods (Bradley and Lang, 1994).

### Acquisition

**Figure 2:**
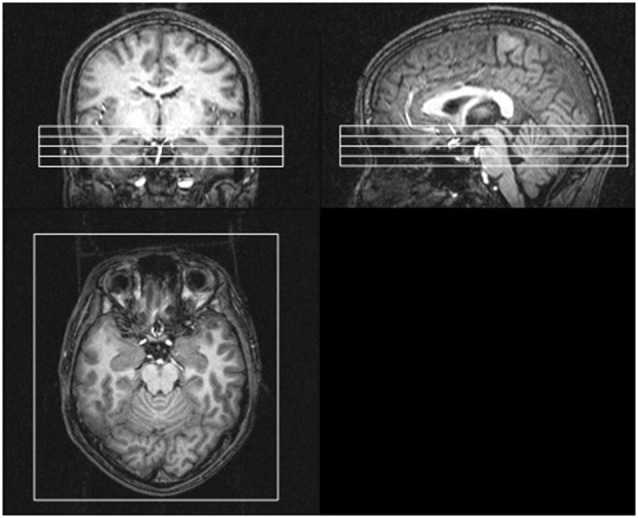
**Positions of four oblique axial slices involving the bilateral amygdalae, the bilateral anterior VLPFCs (BA47) and the primary visual cortex**. The slice position was decided by aligning the top slice with the anterior commissure and the posterior commissure (AC-PC) line.

The picture stimuli were delivered via a goggle system (Resonance Technology, Northridge, CA). The subjects were instructed to look at the crosshair in the middle of the screen, remain still and not fall asleep. The experiments were performed using a Philips Achieva 3.0 T MRI scanner using gradient echo planar imaging, and 1,230 volumes per run were obtained from four oblique axial slices including the bilateral amygdalae, the bilateral anterior VLPFCs (BA47) and the primary visual cortex (V1). The slice position was decided by aligning the top slice with the anterior commissure and the posterior commissure (AC-PC) line (Figure 2).

The acquisition parameters were as follows: repetition time = 300 ms, echo time = 30 ms, flip angle = 40°, field of view = 230 mm, matrix = 128 × 80, number of slices = 4, slice thickness = 5 mm, slice gap = 1 mm, interleaved acquisition. The flip angle parameter was adjusted to maximize the MRI signals from gray matter under the condition of the fast repetition time (300 ms). This run was repeated three times. In addition, high-resolution whole-brain anatomical images were acquired using three-dimensional T1-weighted sequence (repetition time = 7.8 ms, echo time = 3.8 ms, inversion time = 975.8 ms, field of view = 240 mm, matrix = 256 × 240, number of slices = 160, slice volume = 60 mm, slice thickness = 1 mm).

### Data processing

**Figure 3:**
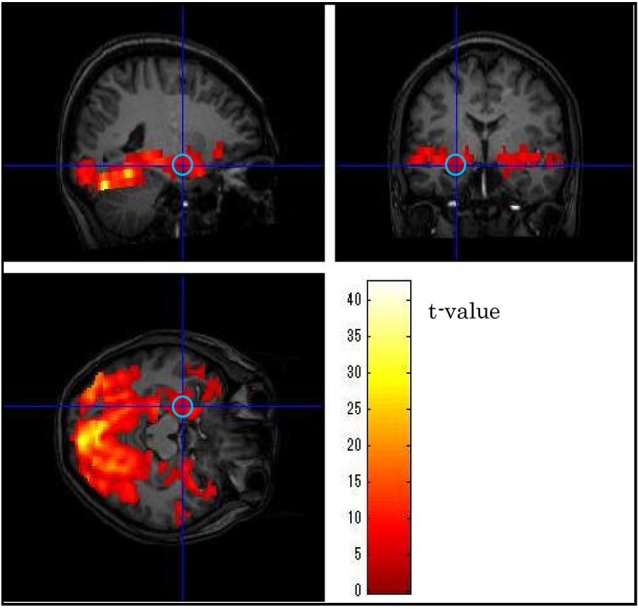
**Example of the position of a volume of interest (left amygdala) in one subject**. The central position was determined using as guides neuroanatomical atlases and three-dimensional activation maps.

We identified the activation maps of the fMRI data by using SPM8 software.^1^ To detrend and analyze the fMRI time-course data, we used a multiple linear-regression algorithm based on the general linear model and then used spatial smoothing with a Gaussian filter (full width at half maximum = 8 mm). All the MRI data were analyzed with a canonical hemodynamic response function (Friston et al., 1998) identical to the activation time course, and an appropriate threshold value (uncorrected, *p* < 0.05) was used for the generation of *t* maps. In each subject, the centers of the VOIs were set to the coordinates for peak *t* values in the bilateral anterior VLPFCs, the bilateral amygdalae and the primary visual cortex responding to both unpleasant and neutral picture stimuli. BOLD signals were averaged over a VOI with a 5-mm radius (Figure 3), and the average value in each brain region was extracted from the fMRI data by using the MarsBar tool for SPM.^2^ Although our objective was to investigate the timing of emotional discrimination in the bilateral VLPFCs and the bilateral amygdalae, the BOLD signal in the primary visual cortex was also extracted in order to verify the validity of the results.

The BOLD signals for each run were linearly detrended. The baseline levels for the signals were set by the averaging in the 3.0 s period (10 points) just prior to the first stimulation epoch in order to acquire baseline signals after steady-state magnetism, and the percent signal changes were calculated. Then the signals were folded into a single epoch by averaging the unpleasant stimulus epochs and the neutral stimulus epochs in each run. Here we confirmed that the differences of the signal changes between unpleasant and neutral picture stimuli at stimulus onset were very small (less than 0.1%). Thus the folded plots were set to zero at the stimulus onset in order to compare the response time courses of the BOLD signals activated by unpleasant and neutral picture stimuli (Kohno et al., 2009).

### Statistical analysis

**Figure 4:**
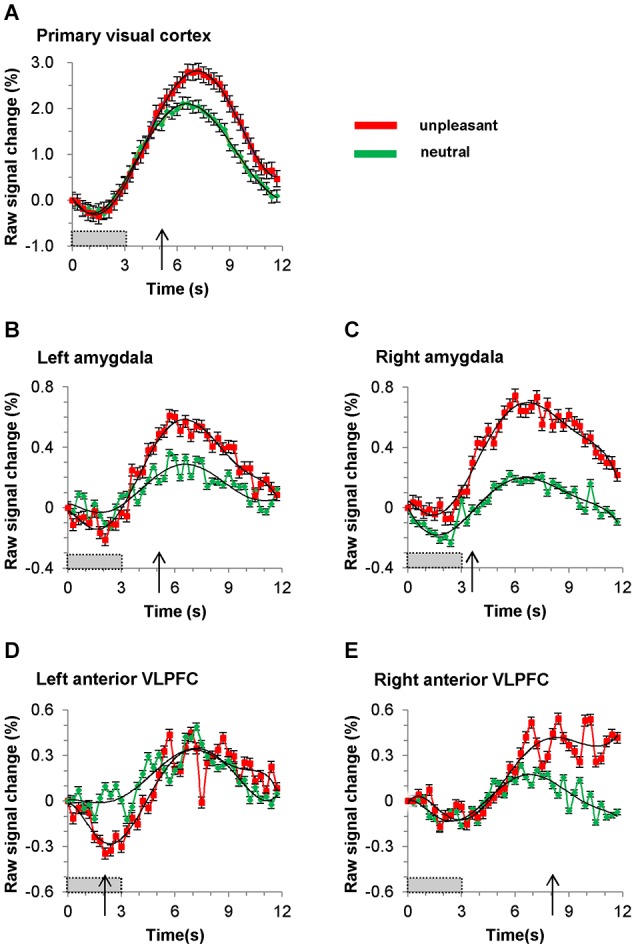
**Time course of BOLD signal in (A) primary visual cortex, (B) left amygdala, (C) right amygdala, (D) left anterior VLPFC (BA47) and (E) right anterior VLPFC (BA47)**. Red and green plots represent averaged signals (for all nineteen subjects) elicited by unpleasant and neutral pictures, respectively. The gray bars show the picture presentation period, and the arrows show the time at which the BOLD signal during presentation of an unpleasant pictures becomes significantly greater than that during presentation of a neutral picture. The black curved lines are sixth-order polynomial fits, and the error bars show standard deviation.

To reliably identify the timing (defined in this paper as the *time after stimulus onset*) of the BOLD signals of emotional discrimination between unpleasant and neutral picture stimuli in the bilateral amygdalae, the bilateral anterior VLPFCs and the primary visual cortex, paired-samples permutation *t*-tests (a straightforward way to solve the multiple-comparisons problem) were performed for each time point in the folded plots (Figure 4; Blair and Karniski, 1993; Maris and Oostenveld, 2007). The *p* values at each time point were calculated from the permutation distribution (number of permutations = 2^19^ = 524,288). The time of emotional discrimination was defined as the first time after stimulus onset at which the *p* value was less than five percent for more than two successive points. To find the data trends, negative peak and positive peak, we used the least-squares method to fit a sixth-order polynomial to the sets of measured data (see black curves in Figure 4).

## Results

### Valence results

The valence ratings of our sample had a mean of 3.08 (*SD* = 0.77) for unpleasant pictures, 5.07 (*SD* = 0.24) for neutral pictures and 6.58 (*SD* = 0.71) for pleasant pictures. The valence rating results in the present study differed significantly between unpleasant and neutral pictures (*p* < 10^−12^; two-sample *t*-test).

### fMRI results

The folded time courses for the unpleasant and the neutral picture stimuli, averaged over all subjects, are shown in Figure 4. The timing of emotional discrimination during negative emotional stimuli is listed in Table 1. Also listed there are the times and percent signal changes of both positive and negative peaks (times and changes calculated from sixth-order polynomial fittings). In our data both the negative peaks and the positive peaks were found in all time courses of BOLD signals in the brain regions. During unpleasant picture presentations the BOLD signal at the positive peak of the response was greater, relative to that at the positive peak during neutral picture presentations, in the primary visual cortex (*p* < 0.001; paired *t*-test), the left amygdala (*p* < 0.05; paired *t-test*), the right amygdala (*p* < 0.001; paired *t-test*) and the right anterior VLPFC (*p* < 0.0001; paired *t-test*). And during unpleasant picture presentations the BOLD signal at the negative peak of the response was greater, relative to that during neutral picture presentations, in the left anterior VLPFC (*p* < 0.01; paired *t-test*). The time courses of positive peaks activated by unpleasant picture stimuli were steeper, relative to those of positive peaks activated by neutral picture stimuli, in the bilateral amygdalae and the left anterior VLPFC, whereas in the primary visual cortex and the right anterior VLPFC those time courses were almost the same.

**Table 1:** **Timing of emotional discrimination in bilateral VLPFCs, bilateral amygdalae and the primary visual cortex**.

Region	Stimulus	Negative peak time (s)/ signal changes (%)	Positive peak time (s)/ signal changes (%)	Timing of emotional discrimination (s)
Primary visual cortex	Unpleasant	1.3/−0.30	7.1/2.8	5.1
	Neutral	1.1/−0.28	6.5/2.1
Left amygdala	Unpleasant	1.6/−0.15	6.5/0.58	5.1
	Neutral	1.8/−0.03	6.6/0.29
Right amygdala	Unpleasant	1.4/−0.04	6.7/0.7	3.6
	Neutral	1.7/−0.18	6.5/0.20
Left anterior VLPFC	Unpleasant	2.4/−0.28	7.1/0.34	2.1
	Neutral	1.8/−0.01	7.2/.35
Right anterior VLPFC	Unpleasant	2.9/−0.12	8.3/0.42	8.1
	Neutral	2.5/−0.13	6.8/0.18

The response for BOLD signals in the primary visual cortex during unpleasant and neutral stimuli showed transient decreases just after stimulus onset. The response dipped after the onset of the visual stimulus, reaching a minimum value of −0.28% at 1.2 s, at which time the response began to recover, and became positive at 2.4 s. Within 4.5 s of the onset of a stimulus there was almost no difference (less than 0.1%) between unpleasant and neutral picture stimuli with regard to the BOLD signals in the primary visual cortex, while the BOLD signals in the bilateral amygdalae and the left anterior VLPFC clearly differed between unpleasant and neutral picture stimuli. After 4.5 s the time course of the BOLD signals in the primary visual cortex differed between unpleasant and neutral picture stimuli.

The timing of emotional discrimination during unpleasant picture stimuli (indicated by *p* < 0.05 for more than two successive points; paired permutation *t*-test) was 2.1 s in the left anterior VLPFC, 3.6 s in the right amygdala, 5.1 s in the left amygdala, 5.1 s in the primary visual cortex and 8.1 s in the right anterior VLPFC.

## Discussion

Investigating the timing of emotional discrimination in the bilateral VLPFCs and the bilateral amygdalae during the viewing of unpleasant pictures, we found that the emotional discrimination in the right amygdala preceded that in the left amygdala and that the emotional discrimination in both these regions preceded that in the right anterior VLPFC.

### Primary visual cortex

The results about the initial BOLD-signal dip in the primary visual cortex were quite consistent with those of previous studies (Duong et al., 2000; Kim et al., 2000; Behzadi and Liu, 2006; Hu and Yacoub, 2012). In addition, the consistency of the early time course (<4.5 s after stimulus onset) between unpleasant and neutral picture stimuli means that the primary visual cortex is not directly involved in the initial emotional discrimination. These results seem to be reasonable.

The late time course (>4.5 s) of V1 BOLD signals differed between unpleasant picture stimuli and neutral picture stimuli. The projections from the amygdala to the primary visual cortex were confirmed using retrograde tracers in macaque monkeys and chimpanzees (Freese and Amaral, 2005, 2006), and the visual emotion discrimination has been thought to result from reentrant feedback from the amygdala to the visual cortex (Iwai and Yukie, 1987; LeDoux, 1998; Herrmann et al., 2008). The BOLD-signal difference between unpleasant and neutral stimuli after 4.5 s is accounted for by the reentrant feedback from the amygdala to the visual cortex because emotional discrimination in the right amygdala preceded that in the primary visual cortex by ~1.5 s.

### Amygdala

A recent meta-analysis of functional imaging studies (Sergerie et al., 2008), unlike some previous studies, did not find evidence for amygdala lateralization in terms of sex or valence but instead provided strong support for a functional dissociation between left and right amygdala in terms of temporal dynamics (Phillips et al., 2001; Wright et al., 2001). In our experiment the emotional discrimination in the right amygdala preceded that in the left amygdala by ~1.5 s. Indeed it has been hypothesized that the right amygdala is part of a rapid or dynamic emotional stimulus detection system and the left amygdala might be specialized for sustained stimulus evaluations (Wright et al., 2001), and our results about the timing of emotional discrimination in the human amygdala support that hypothesis.

### Anterior VLPFC

The left anterior VLPFC (BA47) is responsible for controlled retrieval of semantic information (Wagner et al., 2001; Gold and Buckner, 2002; Velanova et al., 2003; Badre and Wagner, 2007). It should be noted that the subjects in our experiment had to interpret the meaning of the IAPS pictures because the pictures contain various complicated scenes. After that, the meanings of pictures provided to the subjects would be appraised in order to transform a percept into something that elicits emotion (Smith and Ellsworth, 1985). Thus our result that the left anterior VLPFC was implicated in the earliest emotional discrimination (2.1 s) is reasonable because the semantic processing of unpleasant pictures is likely to be a higher perceptual load than the semantic processing of neutral pictures (Eastwood et al., 2001).

The right VLPFC has been characterized as important for response inhibition (Aron et al., 2004; Aron and Poldrack, 2005; Lieberman et al., 2007; Hampshire et al., 2010; Levy and Wagner, 2011; Cohen et al., 2013). A recent meta-analysis has revealed that both middle and posterior subregions of the right VLPFC (i.e., BA45 and BA44) are consistently active during the Go/No-Go and Stop-Signal tasks used to measure the response inhibition, whereas no evidence for the involvement of the right anterior VLPFC (BA47) has been found (Levy and Wagner, 2011). However, it has been hypothesized that the right anterior VLPFC is implicated in the integration of viscerosensory information with affective signals (Lévesque et al., 2004). The integration is supposed to be the last event in the processing of the viscerosensory information. Therefore our result that the emotional discrimination in the right anterior VLPFC occurs after that in the other brain regions investigated in this study supports the above-mentioned hypothesis. Interestingly, unlike the BOLD signals in the other brain regions, those in the right anterior VLPFC remained high after the emotional discrimination of unpleasant picture stimuli. It has been reported that subjective emotional experience involves a relatively long time and that feeling occurs over several tens of seconds (Lutz et al., 2002; Mauss et al., 2005). Alternatively, it is also conceivable that the prolonged right anterior VLPFC activation is due to anticipation (Waugh et al., 2008; Tops et al., 2014). This anticipatory activation would typically be triggered by signs that an aversive stimulus may be imminent. The zigzag pattern in the right anterior VLPFC after the finished emotional stimulus might represent physiological phenomenon. At the moment, there is no valid explanation for the zigzag pattern, however, it should be focused on the future work. Thus the time courses of BOLD signals and the late timing of emotional discrimination in the right anterior VLPFC during the viewing of unpleasant pictures might reflect the mental experience and/or anxious anticipation.

In conclusion, we determined differences in the timing of emotional discrimination in the bilateral anterior VLPFCs and the bilateral amygdalae during the viewing of unpleasant pictures. Emotional discrimination in those brain regions occurred last in the right anterior VLPFC. The present findings imply that the right anterior VLPFC integrates viscerosensory information. They also indicate that the right amygdala is part of a rapid emotional stimulus detection system and the left amygdala is specialized for sustained stimulus evaluation.

## Conflict of interest statement

The authors declare that the research was conducted in the absence of any commercial or financial relationships that could be construed as a potential conflict of interest.

## Footnotes

^1^www.fil.ion.ucl.ac.uk/spm/

^2^http://marsbar.sourcefoge.net/
